# RGS1 serves as an antitumor target to inhibit proliferation of NICN87-DR cells and tumor growth in the gastric cancer mouse model

**DOI:** 10.55730/1300-0152.2616

**Published:** 2022-04-25

**Authors:** Zhixiong CHEN, Banglun LIU, Shouru ZHANG, Lihui CHEN, Yuyu LV, Hao SUN

**Affiliations:** 1 Department of Gastrointestinal Cancer Center, Chongqing University Cancer Hospital, Chongqing, China; 2 Department of Pathology, Chongqing University Cancer Hospital, Chongqing, China

**Keywords:** RGS1, gastric cancer, drug-resistance, proliferation, treatment

## Abstract

Gastric cancer is becoming the 4th leading cause of cancer-associated death worldwide. The purpose of this study was to investigate the role of RGS1 in gastric cancer in vitro and in vivo. Proliferation, migration, invasion, and colony formation of NCIN87 cells and drug-resistant NCIN87 cells (NCIN87-DR) were determined. Cell apoptosis and cell cycle were examined using a flow cytometry assay. RGS1 gene knock-down vector (pLVshshRGS1) and Xenograft tumor mouse model was generated. RGS1 and epithelial-mesenchymal transition (EMT) associated markers, including E-cadherin (E-cad), N-cadherin (N-cad), Slug, and Vimentin were detected using a western blotting assay. Tumor size of Xenograft tumor mouse was measured and Ki67 expression was detected using the immunohistochemical assay. NCIN87-DR cells demonstrated significantly lower proliferation, migration, and invasion compared to those of NCIN87 cells (p < 0.05). NCIN87-DR cells showed obvious early apoptosis and displayed obvious alterations for the cell cycle. NCIN87-DR cells exhibited predominantly higher RGS1 expression than that in NCIN87 cells (p < 0.01). E-cad expression was markedly decreased (p < 0.01) and N-cad (p < 0.05), Slug (p < 0.01), Vimentin (p < 0.05) expressions were significantly increased in NCIN87-DR cells than those in NCIN87 cells. RGS1 gene silence remarkably reduced NCIN87-DR proliferation compared to that in NCIN87-DR cells without treatment (p < 0.01). RGS1 gene-silenced NCIN87-DR cell immunization predominantly inhibited tumor growth in Xenograft tumor mouse than that without RGS1 silence (p < 0.05). RGS1 gene-silenced NCIN87-DR cell immunization significantly downregulated Ki67 expression in tumor tissues compared with that without RGS1 silence. In conclusion, RGS1 gene silence reduced the proliferation of NCIN87-DR cells in vitro and inhibited tumor growth in vivo. Therefore, RGS1 served as an antitumor target for the gastric cancer treatment.

## 1. Introduction

In recent years, gastric cancer is becoming the 4th leading cause of cancer-associated death worldwide, demonstrating a higher incidence in China (Diakowska et al., 2019; Xu et al., 2020). Although epithelial-mesenchymal transition (EMT) is associated with proliferation, migration, and invasion of tumor cells (Tanabe et al., 2015), the specific mechanism of EMT in cancer cells, especially drug-resistant cancer cells, has not been fully clarified. As well known, EMT is a cellular process that demonstrates the transformation from the epithelial-phenotype to the mesenchymal-phenotype, involving the changes of tumor cell migration and invasion (Hou et al., 2018). Normally, cancer cells can freely migrate to different organs when the EMT is involved (Tanabe et al., 2015; Akar et al., 2019). While EMT-mediated cancer migration and invasion usually involve a large number of molecules, such as E-cadherin (E-cad) and N-cadherin (N-cad), both of which are considered to be critical for the proliferation of cancer cells (Tanabe et al., 2014).

Regulators of G-protein signaling (RGS) molecules regulate a variety of signal pathways through activating heterotrimeric G proteins (Moratz et al., 2000). Bioinformatics analysis showed that the regulator of G-protein signaling 1 (RGS1) may be a microRNA target and a negative regulator of G-protein coupled receptor signaling pathway (Zou et al., 2019). Meanwhile, RGS1 can also regulate the aggregation of macrophages in the lesions and protect against inflammatory responses (Patel et al., 2015). RGS1 expression has also been proven to be related to poor prognosis in multiple myeloma (Roh et al., 2017), melanoma (Wang et al., 2019), lung cancer (Dai et al., 2011), and other solid tumors (Sethakorn and Dulin, 2013). Zhu et al. (Zhu et al., 2021) reported that the survival time of gastric cancer patients with higher RGS1 levels was significantly longer than that of patients with lower levels. Additionally, the silence of RGS1 can inhibit inflammation through inactivating proinflammatory factors (Hu et al., 2019), and result in increased responsiveness to the chemoattractants in the human B-lymphoma line (Han et al., 2006). However, to date, only a few studies reported the role of RGS1 in cancer cells, and no studies have even involved the association between RGS1 and EMT of cancer cells.

This study aimed to evaluate the proliferation, migration, and invasion, as well as the expression of EMT associated molecules (RGS1, E-cad, N-cad, Slug, Vimentin) in gastric cells (including normal NCIN87 cells and drug-resistant NCIN87 cells). Effects of RGS1 silence on tumor cell proliferation in vitro and tumor growth in vivo were also evaluated. This study would provide new insight into the improvement mechanism of gastric cancer.

## 2. Materials and methods

### 2.1. Cells and animals

The normal human gastric cancer cell line NCIN87 cells were used in this study. The cells were cultured in DMEM-F12 medium (Gibco, Grand Island, NY, USA) containing 10% fetal bovine serum (FBS, Gibco) and 1% streptomycin-penicillin solution (Beyotime Biotech., Shanghai, China). NCIN87 cells were cultured in a condition of 5% CO_2_ at 37 °C.

For drug-resistant NCIN87 cell line (NCIN87-DR cells), which were generated by treating with trastuzumab (T-mab, a monoclonal antibody applied as a standard treatment for gastric cancer (Wang et al., 2019), purchasing from Herceptin, Genentech, Roche). Briefly, NCIN87 cells in the logarithmic growth stage were digested and subcultured. After NCIN87 cells adhered to the wall, the culture medium was changed into a culture medium containing low concentration trastuzumab (1 μg/mL), and the solution was changed once every 1–2 days. NCIN87 cells were subcultured at 80% fusion. After culturing in low concentration trastuzumab (1 μg/mL) medium for 2–3 generations, NCIN87 cells were cultured by increasing drug concentration (5, 10, 15, 20 μg/mL) in the same way as above. Finally, NCIN87 cells were subcultured in a trastuzumab DMEM-F12 medium containing 20 μg/mL for 2 months, and subsequent detection experiments were conducted.

#### 2.1.1. Animals

Male healthy SPF Balb/c nude mice, aged 6 to 8 weeks and weighing 20 to 25 g, were purchased from Chengdu Dossy Experimental Animals Co. Ltd. (Chengdu, China). Mice were housed in a standard cage with a light/dark cycle of 12 h/12 h and humidity of 45%–50%. Mice also had free access to water and food.

This study has been approved by the Ethical Committee of Chongqing University Cancer Hospital, Chongqing, China (Approved date: Feb. 20th, 2022; Approved No. CZLS2022044-A). All animal procedures were carried out according to the NIH guidelines for Laboratory Animal Care and Safety.

### 2.2. Cell viability determination

The density of NCIN87 cells and NCIN87-DR cells were adjusted to 0.5 × 10^5^ cells/mL and inoculated into 96-well plates. Post culturing for 24 h, 48 h, or 72 h, 10 μL CCK-8 solution (Beyotime Biotech.) was added to 96-well plates and incubated for another 4 h at 37 °C. Then, the absorbance of 96-well plates was detected using a microplate reader (Thermo Fisher Scientific, Hudson, NH, USA) at 450 nm. The cell viability of NCIN87 and NCIN87-DR cells were calculated as the followings: Cell viability = (OD_450_ value of NCIN87/NCIN87-DR group-OD_450_ value of blank group)/(OD_450_ value of negative control group-OD_450_ value of blank group) × 100%. Here, the negative control group was designated as a group using only DMEM-F12 medium.

### 2.3. Cell migration and cell invasion analyses

The transwell assay was conducted as described by a previous study (Yang et al., 2018). For cell migration analysis, 1 × 10^5^/mL NCIN87 or NCIN87-DR cells were suspended with an FBS-free medium and seeded into the upper chamber of a transwell. While a total of 500 μL medium containing 10% FBS was added to the lower chamber of the transwell. Then, NCIN87 cells or NCIN87-DR cells were cultured for 24 h in humidified conditions and 5% CO_2_ at 37 °C. The migrated NCIN87 cells or NCIN87-DR cells were fixed using 70% methanol (SinoPharm group, Shanghai, China) for 5 min, stained using 0.5% crystal violet (Sigma-Aldrich, St. Louis, Missouri, USA) for 20 min, washed using PBS solution for 3 times, and counted using microscopy (Olympus, Tokyo, Japan). For cell invasion analysis, the upper chamber was pretreated using Matrigel (BD) for 3 h at 37 °C. The other procedures were consistent with the processes of the cell migration analysis. Data are defined as amounts of migrated cells and invasive cells.

### 2.4. Colony formation analysis

About 500 NCIN87 cells or NCIN87-DR cells were seeded into a 6-well plate and cultured in DMEM-F12 medium at 37 °C with 5% CO_2_. Cells were incubated at 37 °C about 10 days. When the larger clones were appeared in 6-well plate, incubation of cells was stopped. Then, mediums were discarded, and cells were washed using PBS solution, fixed using 70% methanol for 10 min and stained using 0.1% crystal violet for 20 min. Eventually, images of the above stained NCIN87 cell clones or NCIN87-DR cell clones in plates were captured and counted.

### 2.5. Cell apoptosis and cell cycle analyses

Cell apoptosis and cell cycle were analyzed as a former study (Zhang et al., 2018) described with a few modifications. For apoptosis analysis, NCIN87 cells or NCIN87-DR cells were stained with Annexin V-FITC and PI (BD biosciences, Franklin Lakes, NJ, USA) in dark and immediately detected using Beckman Coulter CytoFLEX flow cytometry (Beckman Coulter Inc., Brea, CA, USA). The percentage of apoptotic cells was quantified as instructed by the manufacturer of flow cytometry. Cells percentage “Q1-LR” was assigned as early apoptosis and “Q1-UR” was assigned as late apoptosis. For cell cycle analysis, NCIN87 cells or NCIN87-DR cells were firstly harvested post 24 h culture, and the cell suspension was then digested. Cells were fixed using ethanol (75%) overnight at 4 °C and centrifuged at 1000 r/min for 10 min. The obtained supernatant was discarded, followed by incubating with PI solution (BD Biosciences) containing RNA enzyme for 4 h at 37 °C in dark. Post washing with PBS, cell cycle was examined with CytoFLEX flow cytometry (Beckman Coulter Inc.) at 488 nm. Data were analyzed using FCAP Array software (BD Biosciences).

### 2.6. Generation of RGS1 shRNAs (pLVshRGS1)

The pLVshRAN-EGFP(2A)-Puro lentiviral vector was used to generate pLVshRGS1 vector. shRNA nucleotide sequences targeting RGS1 were listed as the followings: sense: 5’-GATCCACTTCCGCACTCGAG AATCTATCAAGAGTAGATTCTCGAGTGCGGAAG TTTTTG-3’;antisense:5’-AATTCAAAAACTTCCGCA CTCGAGAATCTACTCT TGATAGAT TCTC GAGTGCGGAAGTG-3’. In brief, CD47 shRNA sequence was cloned into pLVshRAN-EGFP(2A)-Puro vector for generating pLVshRGS1 vector. Then, the synthesized pLVshRGS1 vector was packaged by mixing with psPAX2 and pMD2.G. Total of 1 × 10^5^ NCIN87 cells or NCIN87-DR cells were transfected with siRNA-encoding lentivirus (pLVshRGS1, MOI = 80) in presence of polybrene at a concentration of 1 μg/mL overnight. Subsequently, NCIN87 cells or NCIN87-DR cells were cultured in a medium containing 10% FBS for 24 h and transfected with pLVshRGS1 viral vector.

### 2.7. Real-time PCR

Total RNA was extracted using a Trizol kit and cDNA was synthesized from the extracted RNA using Hifair II 1st strand cDNA synthesis kit (Model: 11121ES60, Yeasen, Shanghai, China) in accordance with the protocol of the manufacturer. The specific primers were synthesized and sequences are listed in Table. The obtained cDNA was amplified by real-time PCR using Hieff UNICON Universal Blue Qpcr SYBR Green Master Mix (Model: 11184ES03, Yeasen, Shanghai, China) as instructed by the manufacturer. Post 40 cycle of PCR reactions, annealing was carried out (95 °C for 10 s, 60 °C for 30 s, and 95 °C for 10 s), and fluorescence signals were collected by means of AB Step One plus Real Time PCR System (Applied Biosystems, Foster city, CA, USA). Relative RGS1 expression was verified using 2^−ΔΔCT^ method, with the GAPDH as a reference gene.

### 2.8. Xenograft tumor model generation and tumor measurement

Mice were randomly divided into 6 groups, including NCIN87 group, NCIN87+T-mab group, NCIN87-DR group, NCIN87-DR+T-mab group, NCIN87-DR+pLVshRGS1 group, NCIN87-DR+pLVshRGS1+Tmab group (n = 6 per group). NCIN87 cells or NCIN87-DR cells (1.5 × 10^6^ cells/mouse) suspended in DMEM-F12 medium were subcutaneously injected into right axilla of mice. When tumor size achieved to 300 mm^3^ (assigned as 0 week), T-mab (5 μg/g body weight) was administrated weekly to each mouse for NCIN87+Tmab group, NCIN87-DR+T-mab group, and NCIN87-DR+pLVshRGS1+T-mab group. While saline was administrated weekly for mice in NCIN87 and NCIN87-DR group. For mice in NCIN87-DR+pLVshRGS1 and NCIN87-DR+pLVshRGS1+T-mab group, NCIN87-DR cells were transfected with pLVshRGS1 virus followed by cell injection into mice. Tumor size (volume) and body weight of mice were recorded each week post the above treatments (from 1st week to 6th week). Tumor volume was calculated as the following formula illustrated: tumor volume (mm3) = (long diameter × short diameter^2^) × 0.52. All above mice were euthanized at 6 weeks posttreatments, and the tumors were isolated and measured.

### 2.9. Immunohistochemical analysis

Tumor samples were resected and isolated from mice, fixed using 4% formaldehyde (Sangon Biotech. Co. Ltd., Shanghai, China), dehydrated using graded ethanol (including 75%, 85%, 95%, and 100% ethanol), treated with xylene, embedded in paraffin, and sectioned into slices (5 μm thickness). Then, paraffin tumor sections were stained using rabbit antimouse Ki-67 antibody (HuaBio., Hangzhou, China.), followed by staining with HRP-labeled goat antirabbit IgG (Beyotime Biotech.). Finally, sections were sealed with neutral gum, and pictures were captured with a microscope (Model: DM4000B, Leica, Germany).

### 2.10. Western blotting analysis

Total proteins were separated from NCIN87/NCIN87-DR cells or tumor tissues using 1 mL of radio immune precipitation assay (RIPA) buffer. The above samples were then immediately sonicated for 2 min to break cell membranes and were centrifuged at 12000 r/min for 5 min at 4 °C. Supernatants containing targeting proteins were collected for western blotting analyses. Concentrations of isolated proteins were examined using a BCA Protein Detection kit (Beyotime Biotech.) as described by the manufacturer. Then, proteins were separated using 10% SDS-PAGE and electro-transferred onto a PVDF membrane. Proteins on PVDF membranes were washed using TBST, blocked using 5% nonfat milk for 1 h at room temperature, and incubated using rabbit antimouse RGS1 (ZEN Bio., Chengdu, China), mouse antimouse E-cadherin (ZEN Bio.), rabbit antimouse N-cadherin (ZEN Bio.), rabbit antimouse Slug (ZEN Bio.), mouse antimouse Vimentin (ZEN Bio.) and rabbit antimouse β-actin antibody (Abcam Biotech., Cambridge, MA, USA) at 4 °C overnight. Next, PVDF membranes were washed using PBST and incubated using HRP-conjugated goat antirabbit IgG (Beyotime Biotech.) or HRP-conjugated goat antimouse IgG (Beyotime Biotech.) at room temperature for 1 h. Finally, the immunoblot bands were visualized with the enhanced chemiluminescence (ECL) kit (Beyotime Biotech.) according to the manufacturer’s instructions.

### 2.11. Statistical analysis

Statistical analyses were carried out using SPSS 21.0 software (IBM Corp., Armonk, NY, USA). Data are presented as mean ± SD. Comparisons between 2 groups were done using Student’s t-test, while comparisons among multiple groups were done with one-way ANOVA followed by Tukey’s post hoc test. The threshold for statistical significance was set as p < 0.05.

## 3. Results

### 3.1. Drug-resistant NCIN87 cells demonstrated lower proliferation, migration, and invasion

**Figure 1:**
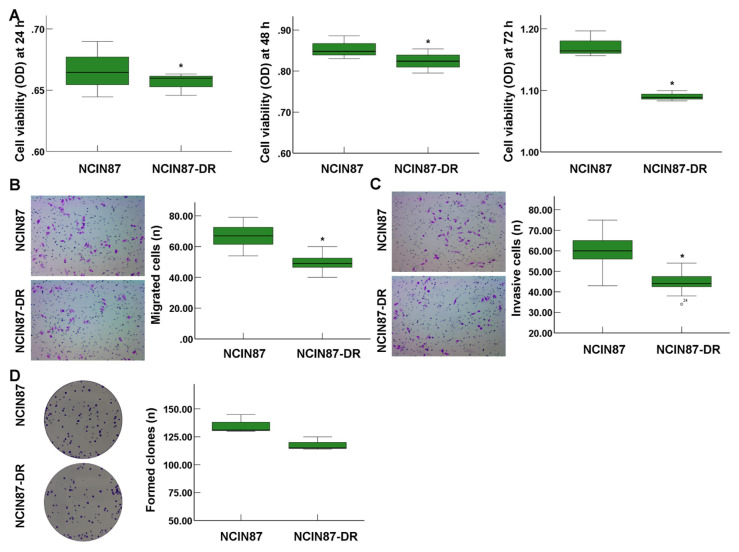
Proliferation characteristics of NCIN87 cells and drug-resistant NCIN87 cells (NCIN87-DR) (n = 3). A. Cell proliferation of NCIN87 and NCIN87-DR cells determined using CCK-8 assay. B. Migration of NCIN87 and NCIN87-DR cells determined using transwell assay. C. Invasion of NCIN87 and NCIN87-DR cells determined using transwell assay. D. Colony formation determined in NCIN87 and NCIN87-DR cells using colony formation analysis. ^*^p < 0.05 versus NCIN87 cells.

In this study, we verified and compared proliferation, migration, and invasion between NCIN87 cells and NCIN87-DR cells. CCK-8 assay findings showed that cell viability of NCIN87-DR cells was significantly lower compared to that of NCIN87 cells at 24 h, 48 h, and 72 h, respectively (Figure 1A, all p < 0.05). Migrated cell accounts (Figure 1B) and invasive cell accounts (Figure 1C) in NCIN87-DR cells were remarkably less than those in NCIN87 cells (all p < 0.05). Moreover, NCIN87-DR cells also demonstrated less colony formation ability compared with that of NCIN87 cells, but without a statistical difference (Figure 1D, p > 0.05).

### 3.2. Drug-resistant NCIN87 cells showed obvious early apoptosis

**Figure 2:**
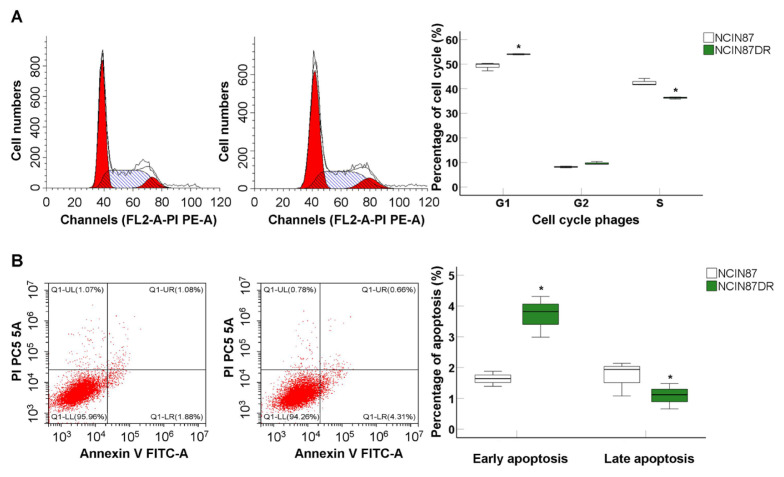
Cell cycle and cell apoptosis were analyzed using flow cytometry (n = 3). A. Cell cycle analysis and statistical analysis for different phage cells. B. Cell apoptosis analysis and statistical analysis for both NCIN87 and NCIN87-DR cells. ^*^p < 0.05 versus NCIN87 cells.

Cell cycle results indicated that there were more G1 phase cells and fewer S phase cells in NCIN87-DR cells compared to those in NCIN87 cells (Figure 2A, all p < 0.05), suggesting that more cells were discovered to be arrested in G0/G1 phase in both NCIN87-DR cells. Flow cytometry assay verified that NCIN87-DR cells showed a higher early apoptosis rate and lower late apoptosis compared to those in NCIN87 cells (Figure 2B, all p < 0.05). Thus, the lower proliferation, migration, and invasion of NCIN87-DR cells might be associated with the changed cell cycle phase and apoptosis.

### 3.3. Drug-resistant NCIN87 cells highly expressed RGS1

**Figure 3:**
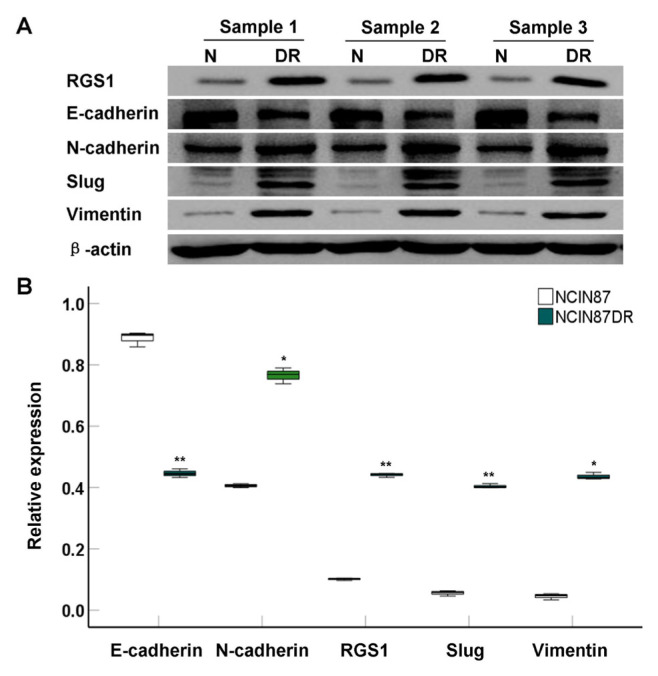
Determination for the RGS1 and EMT-associated molecules expression in both NCIN87 and NCIN87-DR cells (n = 3). A. Western blotting images for RGS1, E-cadherin, N-cadherin, Slug, and Vimentin expression. B. Statistical analysis and comparison for above molecules. ^*^p < 0.05 and ^**^p < 0.01 versus NCIN87 cells.

Western blotting analyses were utilized for determining the expression of EMT-associated molecules, including RGS1, E-cad, N-cad, Slug and Vimentin (Figure 3A). The results illustrated that NCIN87-DR cells exhibited predominantly higher expression of RGS1 compared to that in NCIN87 cells (Figure 3B, p < 0.01). Furthermore, E-cad expression was markedly decreased (p < 0.01) and N-cad (p < 0.05), Slug (p < 0.01), Vimentin (p < 0.05) expressions were significantly increased in NCIN87-DR cells compared to those in NCIN87 cells (Figure 3B). Therefore, EMT might be involved in the drug resistance of NCIN87 cells.

### 3.4. Silence of RGS1 gene reduced proliferation of NCIN87-DR cells

**Figure 4:**
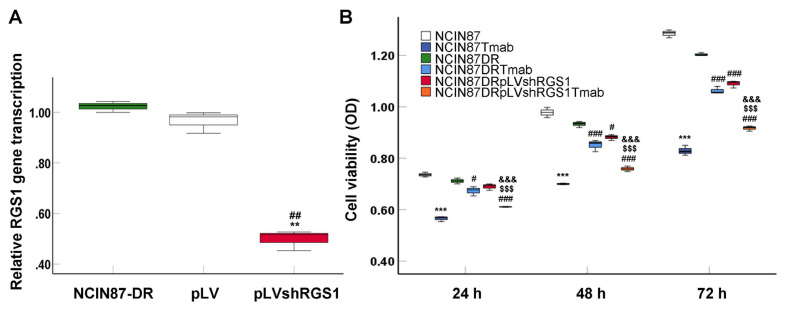
Effects of pLVshRGS1 and/or T-mab treatment on proliferation of NCIN87-DR cells (n = 3). A. RGS1 gene transcription verified with real time PCR assay. ^**^p < 0.01 versus NCIN87-DR group, ^##^p < 0.001 versus pLV group. B. Proliferation evaluation of NCIN87-DR cells using CCK-8 assay and statistical analysis. ^***^p < 0.001 versus NCIN87 group, ^###^p < 0.001 and ^#^p < 0.05 versus NCIN87-DR group, ^$$$^p < 0.001 versus NCIN87-DR+T-mab group, ^&&&^p < 0.001 versus NCIN87-DR+pLVshRGS1+T-mab group.

PCR findings showed that RGS1 gene transcription in pLVshRGS1 transfected NCIN87-DR cells was remarkably lower compared to that in NCIN87-DR cells and pLV blank vector-transfected NCIN87-DR cells (Figure 4A, p < 0.01). Thus, pLVshRGS1 was used for silencing RGS1 gene in NCIN87-DR cells. CCK-8 assay findings showed that tumor-specific antibody T-mab could obviously decrease cell viabilities of NCIN87 cells (p < 0.001) and NCIN87-DR cells (p < 0.05) compared to those cells without T-mab treatments, at 24 h, 48 h, and 72 h post cell culture (Figure 4B). pLVshRGS1 transfection significantly decreased cell viabilities of NCIN87-DR cells when compared with those of NCIN87-DR cells without treatments, at 48 h (p < 0.05) and 72 h (p < 0.001) post culture (Figure 4B). Moreover, NCIN87-DR cells treated with both T-mab and pLVshRGS1 (NCIN87-DR+pLVshRGS1+T-mab group) demonstrated lower cell viability compared with that of NCIN87-DR cells only treated with T-mab (NCIN87-DR+T-mab group) at all time points (Figure 4B, all p < 0.001), suggesting that pLVshRGS1 transfection enhanced the inhibitive effect of T-mab on NCIN87-DR cell proliferation.

### 3.5. RGS1 gene-silenced NCIN87-DR cell immunization inhibited tumor growth of Xenograft tumor mice

**Figure 5:**
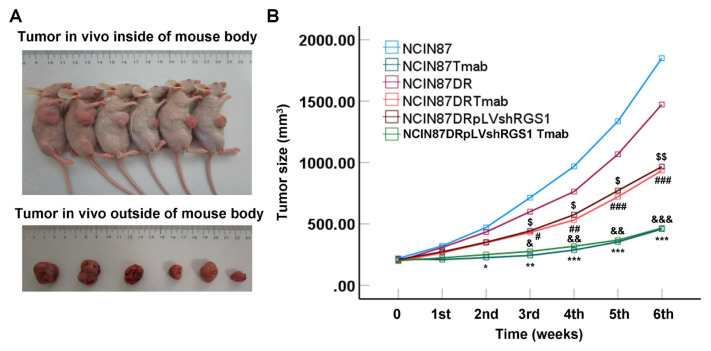
Tumor growth in Xenograft tumor mouse model (n = 3). A. Images of the in vivo tumors in body and isolated tumors. B. Statistical analysis for tumor size. ^***^p < 0.001, ^**^p < 0.01 and ^*^p < 0.05 versus NCIN87 group. ^###^p < 0.001 ^##^p < 0.01 and ^#^p < 0.05 versus NCIN87-DR group, ^$$^p < 0.01 and ^$^p < 0.05 versus NCIN87-DR group, ^&&&^p < 0.001, ^&&^p < 0.01 and ^&^p < 0.05 versus NCIN87-DR+T-mab group.

In vivo tumors in the body and isolated tumors were imaged and measured in RGS1-silenced NCIN87-DR cells immunized mice (Figure 5A). According to measurements of tumors, tumor size of Xenograft mice immunized with T-mab treated NCIN87 cells (T-mab sensitive) was obviously reduced when compared with that of mice immunized without T-mab (Figure 5B, p < 0.05). However, for drug-resistant NCIN87 cells (NCIN87-DR cells), T-mab demonstrated relatively less effect on the tumor size of Xenograft mice (Figure 5B). The RGS1 gene-silenced NCIN87-DR cell immunization obviously decreased the tumor size of Xenograft mice compared to that of solely NCIN87-DR cell immunized Xenograft mice (Figure 5B, p < 0.05). Additionally, RGS1 gene-silenced NCIN87-DR cell immunization could also strengthen the inhibitive effect of T-mab on tumor growth (tumor size) when compared with that of mice in NCIN87-DR+T-mab group (Figure 5B, p < 0.05). Totally, RGS1 gene-silenced NCIN87-DR cell immunization obviously inhibited tumor growth of Xenograft tumor mice.

### 3.6. RGS1 gene-silenced NCIN87-DR cell immunization downregulated Ki67 expression in tumor tissues of Xenograft tumor mice

**Figure 6:**
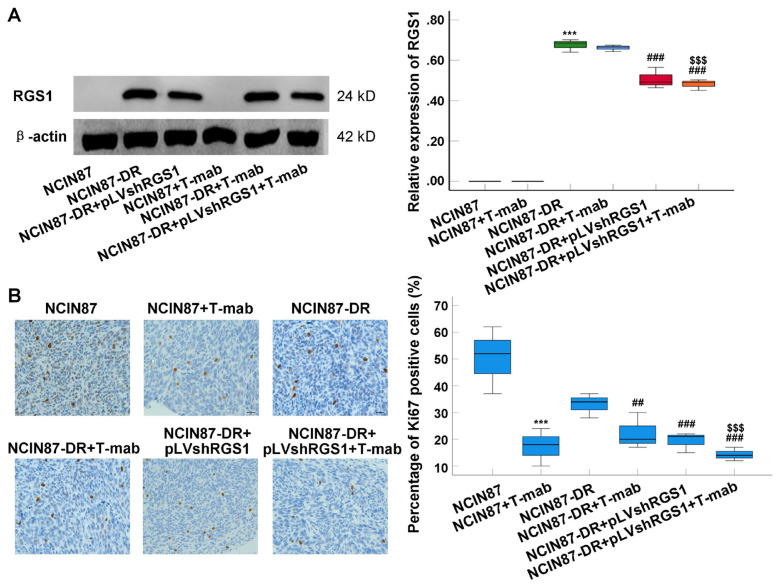
Effects of pLVshRGS1 and/or T-mab treatment on Ki67 expression in tumor tissues of Xenograft tumor mouse model (n = 3). A. Western blotting assay and statistical analysis for RGS1 expression. B. Ki67 expression in tumor tissues using immunohistochemical analysis. ^***^p < 0.001 versus NCIN87 group. ^##^p < 0.01 ^###^p < 0.001 versus NCIN87-DR group. ^$$$^p < 0.001 versus NCIN87-DR+T-mab group.

Western blotting results showed that there was no RGS1 expression in NCIN87 cells (Figure 6A). However, RGS1 expressions in both NCIN87-DR+pLVshRGS1 group and NCIN87-DR+pLVshRGS1+T-mab group were significantly decreased compared with those in NCIN87-DR group (Figure 6A, p < 0.001). The immunohistochemical findings indicated that Ki67 expression was obviously downregulated in tumor tissue of mice in NCIN87-DR+pLVshRGS1 group compared to that in NCIN87-DR group (Figure 6B, p < 0.001). Also, Ki67 expression in tumor tissues of mice in NCIN87-DR+pLVshRGS1+T-mab group was further decreased when compared with that in NCIN87-DR+T-mab group (Figure 6B, p < 0.001). Thus, RGS1 gene-silenced NCIN87-DR cell immunization inhibited tumor growth through downregulating Ki67 expression in tumor tissues of Xenograft tumor mice.

## 4. Discussion

Gastric cancer has become the most common malignancy, with high mortality and incidence (Global Burden of Disease Cancer C, et al., 2017; Chen et al., 2020). Gastric cancer is considered to be the 4th leading cause of cancer-related death in the world (Tan and Yeoh, 2015; Cubiella et al., 2021), therefore, it is urgent to discover a new and effective antigastric cancer strategy. In recent years, immunotherapy, chemotherapy, and radiotherapy have been widely applied in the treatment of various tumors, however, severe side effects are inevitable (Rossi et al., 2012; Kang et al., 2017). Moreover, natural products or reagents extracted from plants or animals also demonstrate high antitumor efficiency and low toxicity (Li et al., 2019). HER2 targeted therapy is a newly developed method to treat HER2 positive tumors in recent years, such as breast cancer (Hackshaw et al., 2020), gastric cancer, and esophageal cancer (Gerson et al., 2017), with obvious effects. However, some drug-resistant cancer cells are usually insensitive to HER2 targeted therapeutic reagents (Yonesaka, 2021). Therefore, this study was conducted to determine the promising therapeutic targets of gastric cancer cells and explore the associated mechanisms.

In this study, we first conducted CCK-8 assay to determine the cell proliferation of NCIN87 cells and NCIN87-DR cells. The results showed that the cell viability of NCIN87-DR cells was significantly lower than that of NCIN87 cells, therefore, NCIN87-DR cells demonstrated lower proliferation. Drug-resistant NCIN87 cells (NCIN87-DR cells) also exhibited lower migration capability and invasion capability. Meanwhile, there were fewer clones formed in NCIN87-DR cells compared with that of NCIN87 cells. Therefore, the proliferation, migration, invasion, and colony formation ability of drug-resistant NCIN87 cells seemed to be low, which is consistent with the findings of a previous study (Zuo et al., 2015). In addition, NCIN87-DR cells displayed obvious cell cycle changes, including the increased G1 phage cells and decrease S phase cells. Drug-resistant NCIN87 cells also showed obvious early apoptosis. Thus, the more slow growth of T-mab resistant NCIN87-DR cells and less proliferation, migrated and invasive capability might be associated with apoptosis and cell cycle.

In addition to migration and invasion, the EMT process of cancer cells is also a key factor in the distant-metastatic cascade of cancer cells (Chen et al., 2020). In fact, the EMT process is related to the migration and invasion of cancer cells (Chen et al., 2020). The damage of EMT process causes the inhibition of migration and invasion (Chen et al., 2020). Therefore, we measured EMT associated biomarkers, including E-cad, N-cad, Slug, and Vimentin, in both NCIN87 cells and NCIN87-DR cells, in order to discover the differentially expressed molecules between two cell lines. Western blotting analysis showed that E-cad expression was markedly decreased and N-cad, Slug, and Vimentin expressions were significantly increased in NCIN87-DR cells compared to those in NCIN87 cells. In fact, a study published by Kim et al. (Kim et al., 2015) has demonstrated that similar changes of EMT associated molecules in drug-resistant cancer cells have been confirmed in breast cancer patients. Therefore, classical EMT associated biomarkers are involved in the drug-resistance of the cancer cells. A previous study (Tanabe et al., 2015) reported that RGS1 plays an important role in tumor cell response and proved the possibility of its involvement in tumor cell immunity. RGS1 protects normal cells from development and progression by increasing anticancer immunity or inhibiting the transformation of cell phenotypes into cancer. Therefore, we speculated that RGS1 might be involved in the transformation of EMT into cancer cells. Western blotting analysis indicated that expression of RGS1 in NCIN87-DR cells was predominantly higher compared to that in NCIN87 cells. RGS1 may participate in the EMT process together with classical EMT related biomarkers and further trigger the drug resistance of NCIN87-DR cells. Although the biofunctions of classical EMT associated biomarkers in cancer progression have been fully clarified (Govindarajan et al., 2010; Lv et al., 2019), it has not been discussed in this study.

In order to determine the role of RGS1 in drug-resistance of NCIN87-DR cells, RGS1-silence vector pLVshRGS1 was generated and transfected into HCIN87-DR cells. CCK-8 assay findings showed that tumor-specific antibody T-mab could obviously decrease cell viability of NCIN87 cells, but had relatively little effect on NCIN87-DR cells. pLVshRGS1 transfection significantly decreased the cell viability of NCIN87-DR cells. Compared with NCIN87-DR cells treated with T-mab, NCIN87-DR cells treated with T-mab combined with pLVshRGS1 demonstrated lower cell viability. These results suggest that pLVshRGS1 transfection enhanced the inhibitive effect of T-mab on the proliferation of drug-resistant NCIN87-DR cells. According to these findings, we found that RGS1 is a target to block the proliferation of cancer cells, which may be beneficial to the function of antitumor drugs (such as T-mab).

We also immunized animals with NCIN87-DR cells to generate a Xenograft tumor mouse model. As the findings show, the tumor size of Xenograft mice immunized with NCIN87 cells treated with T-mab reduced obviously, while the tumor size of Xenograft mice immunized with NCIN87-DR cells treated with T-mab decreased less, showing obvious drug-resistance in vivo. However, RGS1 gene-silenced NCIN87-DR cell immunization obviously decreased tumor size of Xenograft mice compared to that of solely NCIN87-DR cell immunized Xenograft mice. RGS1 gene-silenced NCIN87-DR cell immunization could also strengthen inhibitive effect of T-mab on tumor growth. Thus, RGS1 gene-silenced NCIN87-DR cell immunization could obviously inhibit tumor growth of Xenograft tumor mice. Ki67, as a constitutively expressed molecule in cycling mammalian cells, is widely used to be a cell proliferation biomarker of cancer cells (Sobecki et al., 2016). Therefore, we examined Ki67 expression using an immunohistochemical assay to verify the proliferation of NCIN87-DR cells. The immunohistochemical assay results indicated that RGS1 gene-silenced NCIN87-DR cell immunization could downregulate Ki67 expression in tumor tissues of Xenograft tumor mice. These results reveal that RGS1 gene silence inhibited cancer cell proliferation (NCIN87-DR) and tumor growth through downregulating Ki67 expression. Actually, a previous study (Li et al., 2017) has proven that high expression of RGS20, a member of the RGS family, is correlated with a high percentage of Ki67 expressing cancer cells. Although a former study (Li et al., 2021) reported that high expression of RGS1 was associated with a low differentiation degree of gastric cancer, our study proved for the first time that RGS1 was correlated with expression of Ki67. Totally, the silence of RGS1 could reduce the proliferation of NCIN87-DR cells in vitro and inhibited tumor growth in vivo. These findings are consistent with a previous study (Li et al., 2021) demonstrating that expression of RGS1 is associated with poor differentiation of cancer cells.

This study also demonstrated a few limitations. First, this study silenced the RGS1 gene expression only by treating it with shRGS1 plasmid. In fact, RGS1 gene knock-out mice might be better to illustrate the silence of RGS1 gene. Second, the sample size (mice) was small in this study. Third, the correlation between RGS1 and EMT associated molecules has not been fully clarified. Fourth, this study only conducted PCR assay for determining the silencing effects of pLVshRGS1 on RGS1 expression in NCIN87-DR cells. It is better to confirm these silence effects by western blotting analysis. Fifth, the expression of RGS1 in tumor tissues of Xenograft tumor mice has not been quantified and its correlation with Ki67 has not been analyzed in this study.

## 5. Conclusions

We evaluated the proliferation, migration, and invasion of gastric cancer cells, and determined the expression of RGS1 in NCIN87-DR cells. We also evaluated the antitumor effect of RGS1 gene silence on NCIN87-DR cells in vivo and in vitro. It was found that RGS1 gene silence reduced the proliferation of NCIN87-DR cells and inhibited tumor growth. Overall, this study showed that RGS1 might be an antitumor target for the treatment of gastric cancer.

## Figures and Tables

**Table:** Specific primers for the real time PCR assay.

Genes		Sequences
GAPDH	Forward	AGAAGGCTGGGGCTCATTTG
GAPDH	Reverse	AGGGGCCATCCACAGTCTTC
RGS1	Forward	TCGAGAATCGACAGCCAAGAA
RGS1	Reverse	TAAAGTATTTGCCTGAAGGTCATTT

## References

[b1-turkjbiol-46-4-277] Akar RO Selvi S Ulukaya E Aztopal N 2019 Key actors in cancer therapy: epigenetic modifiers Turkish Journal of Biology 43 3 155 170 10.3906/biy-1903-39 31320814 PMC6620032

[b2-turkjbiol-46-4-277] Chen Y Wu X Liu C Zhou Y 2020 Betulinic acid triggers apoptosis and inhibits migration and invasion of gastric cancer cells by impairing EMT progress Cell Biochemistry and Function 38 6 702 709 10.1002/cbf.3537 32283563 PMC7496801

[b3-turkjbiol-46-4-277] Dai J Gu J Lu C Lin J Stewart D 2011 Genetic variations in the regulator of G-protein signaling genes are associated with survival in late-stage non-small cell lung cancer PLoS One 6 e21120 10.1371/journal.pone.0021120 21698121 PMC3117866

[b4-turkjbiol-46-4-277] Diakowska D Nienartowicz M Grabowski K Rosińczuk J Krzystek-Korpacka M 2019 Toll-like receptors TLR-2, TLR-4, TLR-7, and TLR-9 in tumor tissue and serum of the patients with esophageal squamous cell carcinoma and gastro-esophageal junction cancer Advances in Clinical and Experimental Medicine 28 4 515 522 10.17219/acem/87012 29968427

[b5-turkjbiol-46-4-277] Gerson JN Skariah S Denlinger CS Astsaturov I 2017 Perspectives of HER2-targeting in gastric and esophageal cancer Expert Opinion on Investigational Drugs 26 5 531 540 10.1080/13543784.2017.1315406 28387541 PMC5563845

[b6-turkjbiol-46-4-277] Global Burden of Disease Cancer Collaboration Fitzmaurice C Allen C Barber RM Barregard L 2017 Global, regional, and National Cancer Incidence, mortality, years of life lost, years lived with disability, and disability-adjusted life-years for 32 cancer groups, 1990 to 2015: a systematic analysis for the global burden of disease study JAMA Oncology 3 4 524 548 10.1001/jamaoncol.2016.5688 27918777 PMC6103527

[b7-turkjbiol-46-4-277] Govindarajan R Chakraborty S Johnson KE Falk MM Wheelock MJ 2010 Assembly of connexin43 into gap junctions is regulated differentially by E-cadherin and N-cadherin in rat liver epithelial cells Molecular Biology of the Cell 21 23 4089 4107 10.1091/mbc.E10-05-0403 20881055 PMC2993739

[b8-turkjbiol-46-4-277] Hackshaw MD Danysh HE Singh J Ritchey ME Ladner A 2020 Incidence of pneumonitis/interstitial lung disease induced by HER2-targeting therapy for HER2-positive metastatic breast cancer Breast Cancer Research and Treatment 183 1 23 39 10.1007/s10549-020-05754-8 32591987 PMC7376509

[b9-turkjbiol-46-4-277] Han JI Huang NN Kim DU Kehrl JH 2006 RGS1 and RGS13 mRNA silencing in a human B lymphoma line enhances responsiveness to chemoattractants and impairs desensitization Journal of Leukocyte Biology 79 6 1357 1368 10.1189/jlb.1105693 16565322

[b10-turkjbiol-46-4-277] Hou M Bao X Luo F Chen X Liu L 2018 HMGA2 Modulates the TGFβ/Smad, TGFβ/ERK and Notch Signaling Pathways in Human Lens Epithelial-Mesenchymal Transition Current Molecular Medicine 18 2 71 82 10.2174/1566524018666180705104844 29974827

[b11-turkjbiol-46-4-277] Hu X Tang J Zeng G Hu X Bao P 2019 RGS1 silencing inhibits the inflammatory response and angiogenesis in rheumatoid arthritis rats through the inactivation of toll-like receptor signaling pathway Journal of Cellular Physiology 234 11 20432 20442 10.1002/jcp.28645 31012109

[b12-turkjbiol-46-4-277] Kang YK Boku N Satoh T Ryu MH Chao Y 2017 Nivolumab in patients with advanced gastric or gastro-oesophageal junction cancer refractory to, or intolerant of, at least two previous chemotherapy regimens (ONO-4538-12 ATTRACTION-2): a randomised, double-blind, placebo-controlled, phase 3 trial Lancet 390 10111 2461 2471 10.1016/S0140-6736 17 31827 5 28993052

[b13-turkjbiol-46-4-277] Kim S Lee J Oh SJ Nam SJ Lee JE 2015 Differential effect of EGFR inhibitors on tamoxifen-resistant breast cancer cells Oncology Report 34 3 1613 1619 10.3892/or.2015.4116 26166014

[b14-turkjbiol-46-4-277] Li Q Jin W Cai Y Yang F Chen E 2017 Regulator of G protein signaling 20 correlates with clinicopathological features and prognosis in triple-negative breast cancer Biochemical and Biophysical Research Communications 485 3 693 697 10.1016/j.bbrc.2017.02.106 28237701

[b15-turkjbiol-46-4-277] Li S Yang H Li S Zhao Z Wang D 2021 High expression of regulator of G-protein signalling 1 is associated with the poor differentiation and prognosis of gastric cancer Oncology Letter 21 4 322 10.3892/ol.2021.12584 PMC7933750 33692854

[b16-turkjbiol-46-4-277] Li T Wang N Zhang T Zhang B Sajeevan TP 2019 A systematic review of recently reported marine derived natural product kinase inhibitors Marine Drugs 17 9 493 10.3390/md17090493 31450856 PMC6780990

[b17-turkjbiol-46-4-277] Lv W Wang J Zhang S 2019 Effects of cisatracurium on epithelial-to-mesenchymal transition in esophageal squamous cell carcinoma Oncology Letter 18 5 5325 5331 10.3892/ol.2019.10859 PMC6781646 31612042

[b18-turkjbiol-46-4-277] Moratz C Kang VH Druey KM Shi CS Scheschonka A 2000 Regulator of G protein signaling 1 (RGS1) markedly impairs Gi alpha signaling responses of B lymphocytes Journal of Immunology 164 4 1829 1838 10.4049/jimmunol.164.4.1829 10657631

[b19-turkjbiol-46-4-277] Patel J McNeill E Douglas G Hale AB de Bono J 2015 RGS1 regulates myeloid cell accumulation in atherosclerosis and aortic aneurysm rupture through altered chemokine signalling Nature Communication 6 6614 10.1038/ncomms7614 PMC4374153 25782711

[b20-turkjbiol-46-4-277] Roh J Shin SJ Lee AN Yoon DH Suh C 2017 RGS1 expression is associated with poor prognosis in multiple myeloma Journal of Clinical Pathology 70 3 202 207 10.1136/jclinpath-2016-203713 27445341

[b21-turkjbiol-46-4-277] Rossi A Di Maio M Chiodini P Rudd RM Okamoto H 2012 Carboplatin- or cisplatin-based chemotherapy in first line treatment of small cell lung cancer: the COCIS meta-analysis of individual patient data Journal of Clinical Oncology 30 14 1692 1698 10.1200/JCO.2011.40.4905 22473169

[b22-turkjbiol-46-4-277] Sethakorn N Dulin NO 2013 RGS expression in cancer: oncomining the cancer microarray data Journal of Receptors and Signal Transduction Research 33 3 166 171 10.3109/10799893.2013.773450 23464602

[b23-turkjbiol-46-4-277] Sobecki M Mrouj K Camasses A Parisis N Nicolas E 2016 The cell proliferation antigen Ki-67 organises heterochromatin Elife 5 e13722 10.7554/eLife.13722 26949251 PMC4841783

[b24-turkjbiol-46-4-277] Tan P Yeoh KG 2015 Genetics and molecular pathogenesis of gastric adenocarcinoma Gastroenterology 149 5 1153 1162e3 10.1053/j.gastro.2015.05.059 26073375

[b25-turkjbiol-46-4-277] Cubiella J Pérez Aisa Á Cuatrecasas M Díez Redondo P Fernández Esparrach G 2021 en representación de la Asociación Española de Gastroenterología, la Sociedad Española de Endoscopia Digestiva y la Sociedad Española de Anatomía Patológica. Gastric cancer screening in low incidence populations: Position statement of AEG, SEED and SEAP Gastroenterologia y Hepatologia 44 1 67 86 10.1016/j.gastrohep.2020.08.004 33252332

[b26-turkjbiol-46-4-277] Tanabe S Aoyagi K Yokozaki H Sasaki H 2014 Gene expression signatures for identifying diffuse-type gastric cancer associated with epithelial-mesenchymal transition International Journal Oncology 44 6 1955 1970 10.3892/ijo.2014.2387 24728500

[b27-turkjbiol-46-4-277] Tanabe S Aoyagi K Yokozaki H Sasaki H 2015 Regulated genes in mesenchymal stem cells and gastric cancer World Journal of Stem Cells 7 1 208 222 10.4252/wjsc.v7.i1.208 25621121 PMC4300932

[b28-turkjbiol-46-4-277] Wang DS Liu ZX Lu YX Bao H Wu X 2019 Liquid biopsies to track trastuzumab resistance in metastatic HER2-positive gastric cancer Gut 68 7 1152 1161 10.1136/gutjnl-2018-316522 30269082

[b29-turkjbiol-46-4-277] Wang Y Liu G Ren L Wang K Liu A 2019 Long non-coding RNA TUG1 recruits miR-29c-3p from its target gene RGS1 to promote proliferation and metastasis of melanoma cells International Journal of Oncology 54 4 1317 1326 10.3892/ijo.2019.4699 30720136

[b30-turkjbiol-46-4-277] Xu QH Xiao Y Li XQ Fan L Zhou CC 2020 Resveratrol Counteracts Hypoxia-Induced Gastric Cancer Invasion and EMT through Hedgehog Pathway Suppression Anti-cancer Agents in Medicinal Chemistry 20 9 1105 1114 10.2174/1871520620666200402080034 32238142

[b31-turkjbiol-46-4-277] Yang TY Wu ML Chang CI Liu CI Cheng TC 2018 Bornyl cis-4-Hydroxycinnamate Suppresses Cell Metastasis of Melanoma through FAK/PI3K/Akt/mTOR and MAPK Signaling Pathways and Inhibition of the Epithelial-to- Mesenchymal Transition International Journal of Molecular Science 19 8 2152 10.3390/ijms19082152 PMC6121392 30042328

[b32-turkjbiol-46-4-277] Yonesaka K 2021 HER2-/HER3-Targeting Antibody-Drug Conjugates for Treating Lung and Colorectal Cancers Resistant to EGFR Inhibitors Cancers (Basel) 13 5 1047 10.3390/cancers13051047 33801379 PMC7958627

[b33-turkjbiol-46-4-277] Zhang L Kang W Lu X Ma S Dong L 2018 LncRNA CASC11 promoted gastric cancer cell proliferation, migration and invasion in vitro by regulating cell cycle pathway Cell Cycle 17 15 1886 1900 10.1080/15384101.2018.1502574 30200804 PMC6152531

[b34-turkjbiol-46-4-277] Zhu T Lou Q Shi Z Chen G 2021 Identification of key miRNA-gene pairs in gastric cancer through integrated analysis of mRNA and miRNA microarray American Journal of Translational Research 13 1 253 269 33527022 PMC7847513

[b35-turkjbiol-46-4-277] Zou ZY Liu J Chang C Li JJ Luo J 2019 Biliverdin administration regulates the microRNA-mRNA expressional network associated with neuroprotection in cerebral ischemia reperfusion injury in rats International Journal of Molecular Medicine 43 3 1356 1372 10.3892/ijmm.2019.4064 30664169 PMC6365090

[b36-turkjbiol-46-4-277] Zuo Q Liu J Zhang J Wu M Guo L 2015 Development of trastuzumab-resistant human gastric carcinoma cell lines and mechanisms of drug resistance Scientific Report 5 11634 10.1038/srep11634 PMC4479993 26108989

